# The body mass index change is associated with death or hemodialysis transfer in Japanese patients initiating peritoneal dialysis

**DOI:** 10.1080/0886022X.2022.2163904

**Published:** 2023-01-13

**Authors:** Daiki Kojima, Naoki Washida, Kiyotaka Uchiyama, Eriko Yoshida Hama, Tomoki Nagasaka, Ei Kusahana, Takashin Nakayama, Kengo Nagashima, Yasunori Sato, Kohkichi Morimoto, Takeshi Kanda, Hiroshi Itoh

**Affiliations:** a Department of Endocrinology, Metabolism and Nephrology, Keio University School of Medicine, Tokyo, Japan; b Department of Nephrology, International University of Health and Welfare Narita Hospital, Narita, Japan; c Biostatistics Unit, Clinical and Translational Research Center, Keio University Hospital, Tokyo, Japan; d Apheresis and Dialysis Center, Keio University School of Medicine, Shinjuku, Tokyo, Japan

**Keywords:** Obesity, sarcopenia, frailty, malnutrition, peritonitis, heart failure

## Abstract

A decreased body mass index (BMI) over time is associated with a poor prognosis for patients on hemodialysis. We aimed to examine whether this association also applies to patients with peritoneal dialysis (PD). BMI change was defined as the percentage change in the BMI between the time of PD catheter insertion and six months after its insertion. The association between the BMI change and all-cause mortality or PD discontinuation from six months after PD catheter insertion until October 2021 was investigated. This retrospective cohort study included 122 patients (aged 61.1 ± 12.1 years; 90 males) who underwent PD catheter insertion between January 2008 and March 2020. The median follow-up period was 43.1 (21.2–78.8) months. The median six-month percentage change in the BMI was −2.14 (−5.56–1.84)%, and patients were categorized into tertiles based on their BMI changes. The fully-adjusted Cox regression analysis revealed a significantly higher rate of PD discontinuation or all-cause mortality (hazard ratio (HR): 2.48; 95%; confidence interval (CI): 1.41–4.37) in patients with the lowest tertile (T1, BMI change: < −4.13%) compared to patients with the middle tertile (T2, BMI change: −4.13%–0.67%). The risk was not significantly higher in patients with the highest tertile (T3, BMI change: >0.67%) than those in the T2 group (HR: 1.18; 95% CI: 0.66–2.11). A decreased BMI over time is independently associated with HD transfer or all-cause mortality among patients initiating PD, which highlights the importance of the 6-month BMI change as a novel prognostic marker.

## Introduction

Peritoneal dialysis (PD), one form of renal replacement therapy, is known to be more cost effective and associated with better health-related quality of life (HRQoL) and satisfaction than hemodialysis (HD). Recent systematic reviews have shown that patients on PD had better generic HRQoL (assessed using the 36-Item Short Form Health Survey (SF-36) and European Quality of Life-5 Dimensions (EQ-5D), both of which are instruments for assessing overall health and functions) than patients on HD [1]. PD is widespread in the U.S. and other countries; however, it accounts for only 3% of cases of renal replacement therapy in Japan [2]. However, since Japan is becoming a super-aging society, the proportion of patients on PD is expected to increase in the future from the perspective of social issues such as skyrocketing medical costs and the burden of nursing care associated with HD, as well as from the viewpoint that PD can be performed in a way that is suited to one’s lifestyle. As PD may become more common in an aging society like Japan, there is an immediate need to elucidate prognostic factors for the maintenance of PD.

Obesity is a cardiovascular risk factor [3–5]. However, in patients on maintenance HD, it has been reported that obesity comes with an advantage in survival [6–8]. This ‘obesity paradox’ has been investigated, and previous studies have reported that patients with low body mass indexes (BMIs) had a higher all-cause mortality rate among those on maintenance HD [9–11]. This may be related to the recent focus on protein-energy wasting (PEW). PEW is defined as disordered catabolism resulting from metabolic and nutritional derangements in chronic disease states [12]. Patients with chronic kidney disease (CKD) tend to be more prone to PEW due to malnutrition; thus, they tend to lose weight. A previous report in Australia revealed that time-varying measures of BMI were significantly associated with the mortality risk in both patients on HD and those on PD [13]. In elderly patients on HD who lost weight (>5% BMI loss), the hazard ratio of death was higher than in those whose weights remained stable [14]. However, to the best of our knowledge, there have been no previous studies clarifying whether the above relationship between BMI change and mortality in HD also applies to PD.

From these perspectives, we hypothesized that a decrease in the BMI would be associated with worse clinical outcomes, including all-cause mortality, in patients on PD as well as those on HD. We also hypothesized that a decrease in the BMI would increase the rate of PD discontinuation (transfer to HD), as weight loss and decreased activities of daily living would be expected to make it more difficult to perform PD procedures appropriately. Therefore, we assessed BMI change from BMIs measured at the time of PD initiation and 6 months after with the aim of investigating its association with mortality or PD discontinuation, both of which were important outcomes for patients on PD.

## Materials and methods

### Study population

This single-center retrospective cohort study was conducted in Keio University Hospital, Tokyo, Japan. All protocols were approved by the ethics committee of this hospital (approval number: 20221014). The study was conducted according to the principles of the Declaration of Helsinki. We included patients who commenced PD as the initial dialysis treatment in our hospital between January 2008 and June 2020 and gave their consent for participation in and publication of this study. Patients without BMI data at the time of PD catheter insertion and/or 6 months later, including those who withdrew from PD before the end of the 6-month period, were excluded from this study. Patients were also excluded if they were younger than 18 years.

### Data collection and patient evaluation

At the time of PD induction, the following data were extracted from the electronic medical record of our hospital: age, sex, primary disease and complications of end-stage kidney disease, blood pressure in mmHg (including the use of antihypertensive drugs or diuretics), height (m) and weight (kg), inferior vena cava diameter (IVCD) (cm), serum albumin (g/dL), corrected calcium (mg/dL), phosphorus (mg/dL), parathyroid hormone (pmol/L), brain natriuretic peptide (BNP) (pg/ml), hemoglobin (g/dL), C-reactive protein (mg/dL), urea nitrogen (mg/dL), creatinine (mg/dL), and estimated glomerular filtration rate (eGFR; ml/min/1.73 m^2^) calculated using the serum creatinine level by three-variable Japanese equations [15]. The BMI at PD induction (at the time of catheter insertion) and 6 months after PD induction was calculated by dividing body weight (BW) by the square of the body height. The geriatric nutritional risk index (GNRI) was calculated from each patient’s BMI and serum albumin level [16]. The dialysis/plasma creatinine ratio at 4 h (D/P4) was measured by a standard peritoneal permeability test [17]. These anthropometric and biochemical variables were also obtained 6 months after PD induction. The Charlson comorbidity index (CCI) was calculated from the records.

### Follow-up

The participants of this study were categorized into three tertiles based on each one’s BMI change, which is defined as the percentage change in the BMI from the time of PD catheter insertion till 6 months after its insertion. The observation period was basically until either PD discontinuation, death, kidney transplantation, or study completion (October 2021) from 6 months after the initiation of PD. The primary outcome was the time until all-cause mortality or PD discontinuation; namely, complete HD conversion. Because peritonitis and heart failure (HF) due to fluid overload are the two leading causes of PD discontinuation in Japan [18], the secondary endpoints were the time-lapse till the development of peritonitis and HF-related hospitalization.

### Statistical analysis

Continuous variables were presented as means ± standard deviations or medians (interquartile ranges) based on normality as assessed using the Shapiro–Wilk test. Categorical variables were presented as frequencies and percentages (%). Normally and non-normally distributed continuous variables were compared between groups using the one-way analysis of variance and the Kruskal–Wallis test, respectively. Fisher’s exact test was used (for categorical variables) to compare parameters between groups. Survival curves were plotted *via* the Kaplan–Meier method. Those were compared using the log-rank test with the post-hoc Bonferroni correction. Adjusted Cox proportional hazards models were used to determine hazard ratios (HRs) with 95% confidence intervals (CIs) for survival. In addition to the 6-month BMI change, variables that were previously shown to be associated with the time-lapse till HD transfer were included in the multivariate regression model. In addition, according to the ‘10 events per variable rule’ to prevent overfitting, <7 independent variables were selected in model 1 and the number of independent variables increased stepwise from model 1 to 3. Consequently, these analyses were performed using three distinct models as follows: Model 1 was a minimally-adjusted model with age, sex, CCI, eGFR, GNRI, and the categories of the BMI change included as candidate independent variables [19–23], taking the multicollinearity between the CCI and diabetes mellitus (DM) or between the BMI and GNRI into consideration. Model 2 was further adjusted for the mean blood pressure (MBP) with the use of angiotensin-converting enzyme inhibitors/angiotensin II receptor blockers (ACEi/ARBs) [24]. Finally, model 3 was the fully-adjusted model, including logarithmic BNP, which was one of the volume status markers, as an independent variable to minimize the effect of fluid overload on outcomes, considering that the BMI does not distinguish between muscle mass, fat, and fluids [25]. Moreover, the logarithmic CRP level, which was one of the markers of systemic inflammation, or malnutrition-inflammation-atherosclerosis syndrome, was also included as one of the candidate covariates in addition to the same variables as model 2 [26]. For sensitivity analyses, we added model 1′, 2′, and 3′, in which logarithmic BMI and serum albumin level replaced GNRI among the independent variables in models 1, 2, and 3, respectively. Furthermore, we modeled the nonlinear associations between the 6-month BMI change and the primary outcome *via* Cox regression models using restricted cubic splines with four knots (5th, 35th, 65th, and 95th percentiles).

Additionally, as transplantation was a competing risk event against death and HD transfer, the cumulative incidence considering the competing risk was compared using Gray’s test, and the Fine and Gray sub-distribution hazards model was used in the multivariate model as a sensitivity analysis method for the primary outcome, together with the standard Cox regression model for cause-specific hazards [19].

The above statistical analyses were performed using EZR (Saitama Medical Center, Jichi Medical University, Saitama, Japan), a graphical user interface for R (The R Foundation for Statistical Computing, Vienna, Austria) [27], and Stata version 17.0 (Stata Corporation, College Station, TX). A statistically two-sided *p*-value of < 0.05 was considered statistically significant.

## Results

### Patients’ clinical characteristics

**Figure 1.**
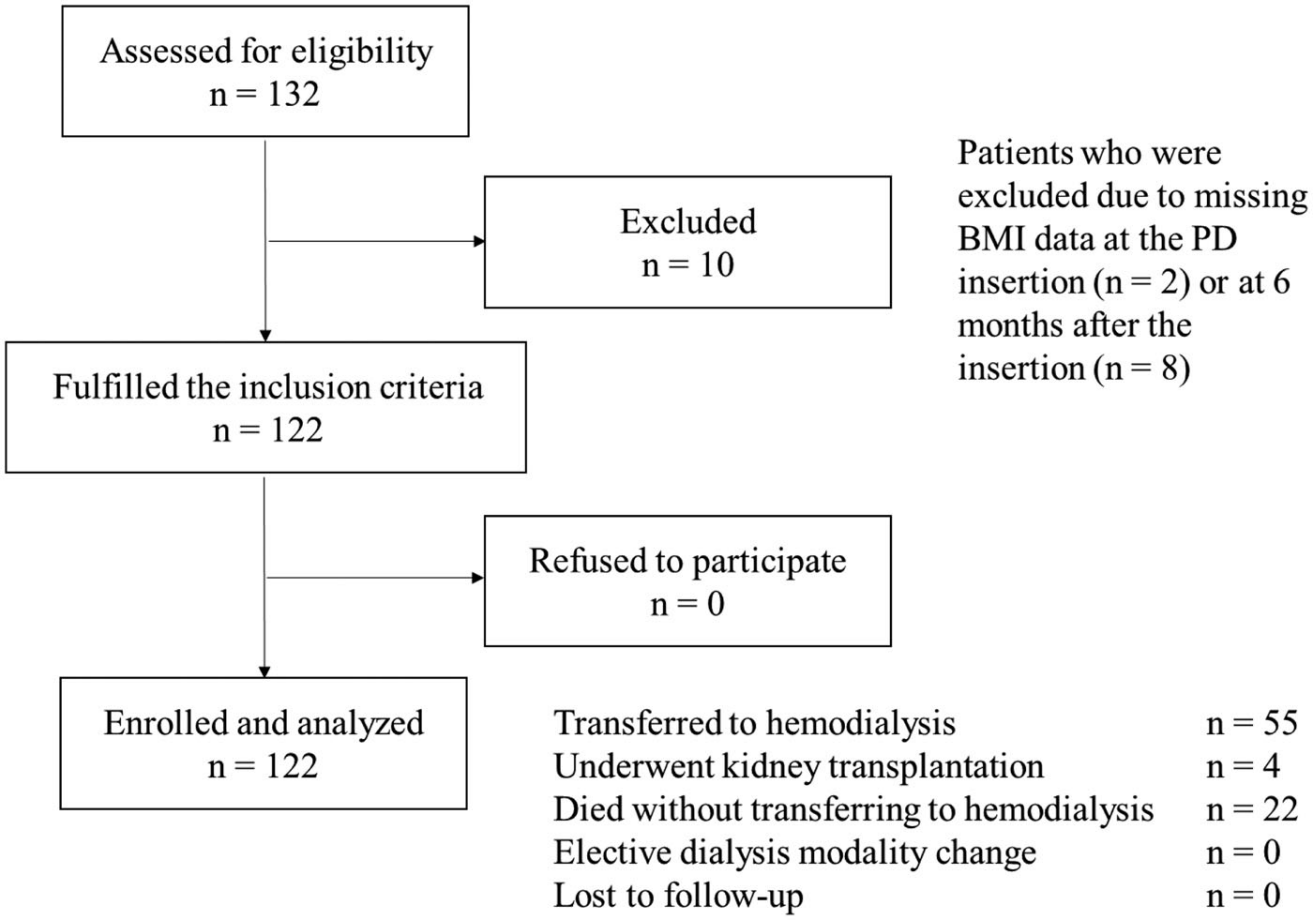
Flow chart of the procedure of study participant recruitment.

Among the 132 eligible patients on PD, ten were excluded because of missing BMI data at the time of PD insertion (*n* = 2) or at 6 months after the insertion (*n* = 8; Figure 1). Baseline patient characteristics are presented in Table 1. The median 6-month percentage change in the BMI was −2.14 (−5.56–1.84)%, and patients were categorized into three tertiles based on each one’s BMI change; the lowest tertile (*n* = 41) (T1, BMI change: < −4.13%), the middle tertile (*n* = 40; T2, BMI change: −4.13%–0.67%) which is the reference group, and the highest tertile (*n* = 41; T3, BMI change: >0.67%). The T1 group exhibited significantly higher baseline BNP levels than in the T2 group (*p* = 0.02). The participants in the T1 group exhibited significantly higher systolic, diastolic, and MBP values than those in the T3 group (*p* = 0.04, 0.04, and 0.02, respectively). Compared with participants in the T2 and T3 groups, those in the T1 group had significantly higher BW and BMI values (*p* = 0.01 and <0.001, respectively). The CRP levels in the T3 group were significantly higher than those in the T2 group. There were no significant differences in the remaining characteristics between the groups. At 6 months, IVCD was significantly larger in the T3 group among the three tertile groups (*p* = 0.03), whereas no significant difference was found in the remaining characteristics, including blood pressure, BNP, and D/P4, among the groups (supplemental Table S1).

**Table 1.** Baseline characteristics of the study population and the groups divided according to body mass index change (T1: BMI change: < −4.13%, T2: BMI change: −4.13–0.67%, and T3: BMI change: >0.67%).

Variables	Total (*n* = 122)	T1 (*n* = 41)	T2 (*n* = 40)	T3 (*n* = 41)	*p*-Value
Age (year)	61.1 ± 12.1	60.8 ± 14.0	59.8 ± 10.7	62.8 ± 11.5	0.53
Sex (% male)	90 (74%)	31 (76%)	30 (75%)	29 (71%)	0.9
APD/CAPD (at the commencement of PD)	0/122 (0/100%)	0/41 (0/100%)	0/40 (0/100%)	0/41 (0/100%)	1
SPIED/SMAP	111/11 (91/9%)	39/2 (95/5%)	34/6 (85/15%)	38/3 (93/7%)	0.27
Double/triple cuffs (Swan neck silicon PD catheter)	101/21 (83/17%)	31/10 (76/24%)	36/4 (90/10%)	34/7 (83/17%)	0.23
Diabetes mellitus	41 (34%)	14 (34%)	13 (33%)	14 (34%)	1
Cerebrocardiovascular disease	32 (26%)	14 (34%)	11 (28%)	7 (17%)	0.22
Antihypertensive drugs					
ACEi/ARB	84 (69%)	33 (80%)	27 (68%)	24 (59%)	0.09
CCB	107 (88%)	34 (83%)	35 (88%)	38 (93%)	0.4
MRB	3 (2%)	1 (2%)	1 (3%)	1 (2%)	1
β-blocker	28 (23%)	10 (24%)	11 (28%)	7 (17%)	0.55
α-blocker	12 (10%)	6 (15%)	4 (10%)	2 (5%)	0.33
Loop diuretics	55 (45%)	20 (49%)	21 (53%)	14 (34%)	0.22
Thiazide diuretics	6 (5%)	2 (5%)	3 (8%)	1 (2%)	0.53
Charlson comorbidity index	3.0 (2.0–4.0)	3.0 (2.0–5.0)	3.0 (2.0–4.0)	3.0 (2.0–4.0)	0.72
Systolic blood pressure (mmHg)	140.1 ± 21.8	146.7 ± 24.9	138.4 ± 16.9	135.0 ± 21.7*	0.04
Diastolic blood pressure (mmHg)	81.8 ± 16.9	86.4 ± 22.1	81.9 ± 10.2	77.0 ± 15.0*	0.04
Mean blood pressure (mmHg)	101.2 ± 17.2	106.5 ± 21.8	100.7 ± 10.9	96.3 ± 15.7*	0.02
Body weight (kg)	65.5 ± 15.3	71.3 ± 16.0	62.3 ± 13.1*	62.8 ± 15.2*	0.01
BMI (kg/m^2^)	23.8 (21.3–26.8)	25.9 (23.7–29.2)	23.0 (21.0–24.9)**	22.7 (20.9–26.4)**	<0.001
IVCD (cm)	1.7 ± 0.4	1.7 ± 0.4	1.6 ± 0.4	1.7 ± 0.3	0.58
Albumin (mg/dL)	3.5 (3.1–3.8)	3.4 (3.1–3.8)	3.6 (3.4–3.9)	3.6 (3.1–3.8)	0.23
Urea (mg/dL)	78.1 (62.8–89.4)	79.2 (60.9–94.5)	73.4 (60.1–85.9)	80.8 (63.2–89.0)	0.72
Creatinine (mg/dL)	8.6 (7.6–9.9)	8.8 (8.0–10.0)	8.6 (7.7–9.9)	8.3 (7.5–9.4)	0.35
eGFR (mL/min/1.73m^2^)	5.4 ± 1.4	5.3 ± 1.7	5.4 ± 1.1	5.4 ± 1.3	0.88
Hemoglobin (g/dL)	9.6 ± 1.3	9.8 ± 1.3	9.6 ± 1.2	9.4 ± 1.5	0.48
Calcium(mg/dL)	8.3 ± 1.1	8.3 ± 1.2	8.4 ± 0.9	8.3 ± 1.0	0.77
Phosphorus (mg/dL)	5.9 (5.1–6.8)	6.0 (5.3–6.9)	5.8 (5.1–6.7)	5.4 (4.8–6.6)	0.16
PTH (pmol/L)	352 (222–500)	298 (201–496)	344 (201–496)	402 (269–521)	0.43
CRP (mg/L)	0.08 (0.03–0.18)	0.10 (0.03–0.17)	0.04 (0.02–0.10)	0.10 (0.05–0.33)^#^	0.01
BNP (pg/mL)	120.5 (50.9–289.5)	166.1 (92.4–413.4)	74.2 (42.5–166.9)*	132.8 (43.4–236.0)	0.02
GNRI	97.3 ± 11.1	99.8 ± 10.9	96.9 ± 10.2	95.3 ± 11.8	0.18
D/P4 (*n* = 118)	0.59 ± 0.13	0.57 ± 0.14	0.57 ± 0.12	0.63 ± 0.14	0.07
6-month percentage change in BMI	−2.14 (−5.57–1.84)	−8.80 (−11.90 to −5.57)	−2.14 (−3.12 to −0.42)***	3.52 (1.93–5.40)^***###^	<0.001

*p < 0.05; **p < 0.01; ***p < 0.001 versus T1. ^#^*p* < 0.05; ^##^*p* < 0.01; ^###^*p* < 0.001 versus T2. *p*-Values were estimated by Fisher’s exact test for categorical variables and the one-way analysis of variance and the Kruskal–Wallis test for normally and non-normally distributed continuous variables, respectively.

Abbreviations: SD: standard deviation; SPIED: short-term PD induction and education; SMAP: stepwise initiation of PD using the Moncrief–Popovich technique; ACEi/ARB: angiotensin converting enzyme inhibitor/angiotensin II receptor blocker; CCB: calcium channel blocker; MRB: mineralocorticoid receptor blocker; BMI: body mass index; IVCD: inferior vena cava diameter; eGFR: estimated glomerular filtration rate; PTH: parathyroid hormone; CRP: C-reactive protein; BNP: brain natriuretic peptide; GNRI: geriatric nutritional risk index; D/P4: dialysate-to-plasma ratio of creatinine at 4 h.

In this study, the median follow-up period was 43.1 (21.2–78.8) months. During follow-up, 55 (45.1%) patients discontinued PD and transferred to HD due to peritonitis (*n* = 29), difficulties in controlling HF due to volume overload (*n* = 8), uremic solute retention (*n* = 4), difficulties in performing PD due to frailty (*n* = 6), major abdominal surgery (*n* = 4), tunnel infection (*n* = 2), catheter malfunction (*n* = 1), and cerebrovascular disease leading to physical disability (*n* = 1) These data can be seen in Table 2. In addition, 22 (18.0%) patients died and 4 (3.3%) patients underwent kidney transplantation.

**Table 2.** Causes of HD transfer among tertile categories.

Cause of HD transfer	Total (*n* = 122)	T1 (*n* = 41)	T2 (*n* = 40)	T3 (*n* = 41)	*p*-Value
Peritonitis	29 (23.8%)	11 (26.8%)	7 (17.5%)	11 (26.8%)	0.16
HF due to volume overload	8 (6.6%)	6 (14.6%)	0 (0%)*	2 (4.9%)	0.04
Uremic solute retention	4 (3.3%)	0 (0%)	3 (7.5%)	1 (2.4%)	0.12
Frailty	6 (4.9%)	3 (7.3%)	2 (5.0%)	1 (2.4%)	0.87
Major abdominal surgery	4 (3.3%)	1 (2.4%)	3 (7.5%)	0 (0%)	0.22
Tunnel infection	2 (1.6%)	1 (2.4%)	1 (2.5%)	0 (0%)	1
Catheter malfunction	1 (0.8%)	0 (0%)	1 (2.5%)	0 (0%)	0.60
Cerebral hemorrhage	1 (0.8%)	0 (0%)	1 (2.5%)	0 (0%)	0.60

*p < 0.05 versus T1. *p*-Values were estimated by Fisher’s exact test.

Abbreviations: HD: hemodialysis; HF: heart failure.

### Association between the BMI change and death or HD transfer in patients on PD

During follow-up, death was observed in 7 (17.1%), 6 (15.0%), and 9 (22.0%) patients in the T1, T2, and T3 groups, respectively, whereas PD discontinuation was observed in 22 (53.7%), 18 (45.0%), and 15 (36.6%) patients, respectively. The causes of HD conversion between the tertile category groups are separately shown in Table 2. The percentage of cases in which HF was the cause of PD withdrawal was significantly higher in the T1 group than the T2 group (*p* = 0.04).

**Figure 2.**
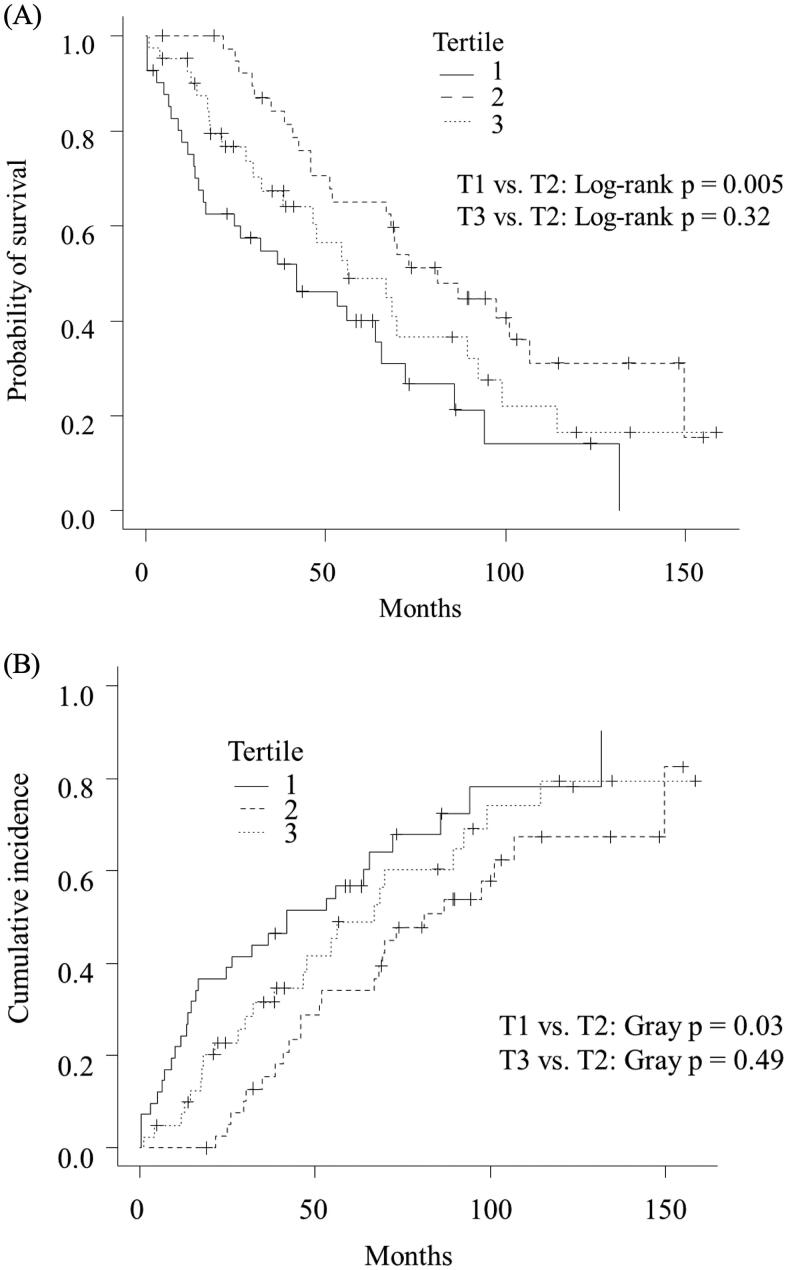
Kaplan–Meier curves (A) and Cumulative incidence curves (B) for death or hemodialysis transfer in the T1, T2, and T3 groups.

The log-rank test showed that the median time-lapse until death and conversion to HD was significantly shorter in the T1 group than in the T2 group (42.0 vs. 81.1 months, *p* = 0.005); however, this time-lapse did not differ significantly between the T2 and T3 groups (81.1 vs. 56.3 months, *p* = 0.32; Figure 2). In addition, the cumulative incidence of death or HD transfer was significantly higher in the T1 group than in the T2 group (*p* = 0.03 using Gray’s test); however, this parameter did not differ significantly between the T2 and T3 groups (*p* = 0.49 using Gray’s test; Figure 2).

The adjusted Cox regression analysis revealed a significantly higher rate of HD transfer or all-cause mortality (HR: 2.48; 95% CI: 1.41–4.37) in patients in the T1 group compared to those in the T2 group, the reference group. The risk was not significantly higher in patients in the T3 group than in those in the T2 group (HR: 1.18; 95% CI: 0.66–2.11; Table 2). The risk was higher among patients in the T1 group than among those in the T2 group in model 2 (HR: 2.53; 95% CI: 1.42–4.50); in contrast, the risk was not significantly higher among patients in the T3 group than among those in the T2 group (HR: 1.18; 95% CI: 0.66–2.12; Table 3). Similarly, in model 3, the risk was persistently higher among patients in the T1 group than among those in T2 (HR: 2.16; 95% CI: 1.19–3.94); however, the risk was not significantly higher among patients in the T3 group than among those in the T2 group (HR: 0.86; 95% CI: 0.46–1.62). In addition, the risk was significantly higher among patients in the T1 group than among those in T3 in model 3 (HR: 2.52; 95% CI: 1.36–4.66), similar to models 1 and 2. Furthermore, in model 3′, higher risk was persistent among patients in the T1 group than among those in the T2 group (HR: 1.92; 95% CI: 1.02–3.65; supplemental Table S2), similar to models 1′ and 2′. The risk was significantly higher among patients in the T1 group than among those in T3, specifically in model 3′ (HR: 2.20; 95% CI: 1.14–4.26). In these multivariate analyses, variance inflation factors for all independent variables that are less than 2, suggesting that there was no multicollinearity. Furthermore, all multivariate models were assumed to hold a proportional hazard assumption and had a sufficiently high concordance index of around 0.70.

**Table 3.** Association between BMI change and death or HD transfer in patients on PD using standard Cox regression model and Fine and Gray sub-distribution hazards model.

HD transfer or all-cause mortality	T1 vs. T2 (ref)			T3 vs. T2 (ref)			T1 vs. T3 (ref)	
	HR (95% CI)	*p*-Value		HR (95% CI)	*p*-Value		HR (95% CI)	*p*-Value
Cause-specific hazards								
Model 1	2.48 (1.41–4.37)	0.002		1.18 (0.66–2.11)	0.57		2.10 (1.18–3.74)	0.01
Model 2	2.53 (1.42–4.50)	0.002		1.18 (0.66–2.12)	0.58		2.14 (1.18–3.87)	0.01
Model 3	2.16 (1.19–3.94)	0.01		0.86 (0.46–1.62)	0.64		2.52 (1.36–4.66)	<0.001
Subdistribution hazards								
Model 1	1.99 (1.20–3.30)	0.008		1.10 (0.65–1.84)	0.73		1.82 (1.01–3.26)	<0.05
Model 2	2.02 (1.20–3.40)	0.008		1.09 (0.65–1.83)	0.74		1.85 (1.01–3.38)	<0.05
Model 3	1.70 (1.02–2.83)	0.04		0.78 (0.43–1.40)	0.41		2.18 (1.16–4.08)	0.02

Model 1 was a minimally adjusted model with age, sex, Charlson comorbidity index, estimated glomerular filtration rate, geriatric nutritional risk index, and the categories of BMI change. Model 2 was adjusted for the same variables as model 1 in addition to the use of the mean blood pressure and angiotensin converting enzyme inhibitor/angiotensin II receptor blocker. Model 3 was adjusted for the same variables as model 2 in addition to the logarithmic brain natriuretic peptide and logarithmic C-reactive protein.

Abbreviations: BMI: body mass index; HD: hemodialysis; PD: peritoneal dialysis; ref: reference; HR: hazard ratio; CI: confidence interval.

**Figure 3.**
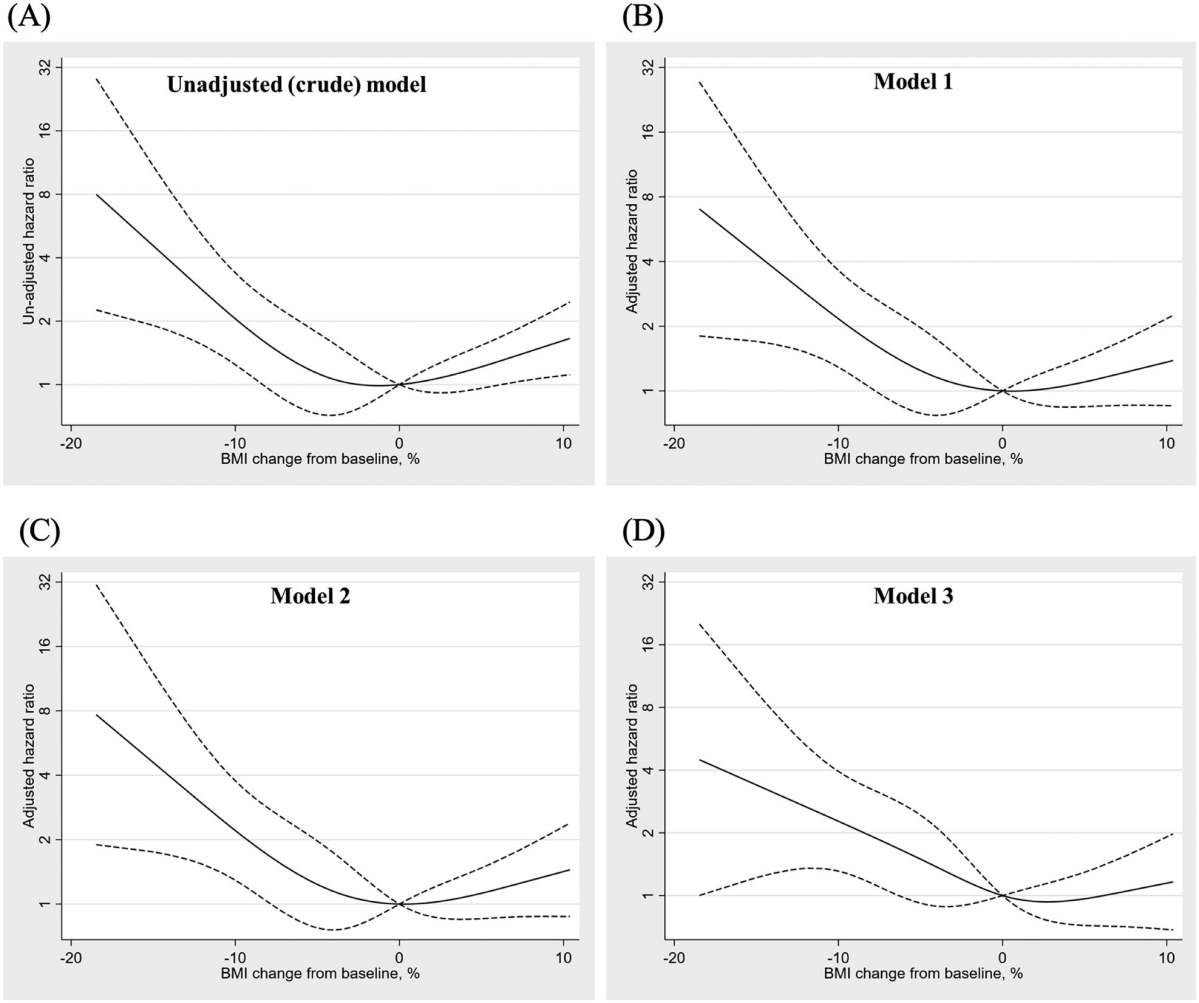
Restricted cubic spline curves showing death or transfer to hemodialysis for the unadjusted (A) and multivariable-adjusted model 1 (B), model 2 (C), and model 3 (D). In splines, solid lines represent the hazard ratio and dashed lines represent the 95% confidence interval.

In the unadjusted and multivariable-adjusted cubic spline analyses evaluating death or HD transfer using the percentage change in the BMI as a continuous variable, reductions in the BMI over a 6-month period were associated with increased mortality or HD transfer (Figure 3).

Adjusted Fine-Gray sub-distributional hazard models, as sensitivity analyses, revealed similar results, including a significantly higher risk of death or HD transfer in the T1 group than in the T2 group, remaining even in the fully-adjusted model 3 (HR: 1.70; 95% CI: 1.02–2.83).

### Association between BMI change on peritonitis and HF in patients on PD

During follow-up, 62 (51.6%) patients developed peritonitis. The median peritonitis-free survival time did not differ significantly between the T1, T2, and T3 groups (35.5 vs. 132.3 vs. 46.7 months, respectively; *p* = 0.15). The adjusted Cox regression analysis revealed that the peritonitis risk was not significantly higher in the T1 group than in the T2 group although borderline in models 1, 2, and 3 (HR: 1.89, 95% CI: 0.98–3.62; HR: 1.85, 95% CI: 0.95–3.61; and HR: 1.79, 95% CI: 0.89–3.60, respectively), whereas it revealed no significant difference between the T2 and T3 groups or between the T1 and T3 groups (Table 4).

**Table 4.** Association between the BMI change and peritonitis or heart failure in patients on PD using the standard Cox regression model.

	Peritonitis									Heart failure-associated hospitalization							
	T1 vs. T2 (ref)			T3 vs. T2 (ref)			T1 vs. T3 (ref)			T1 vs. T2 (ref)			T3 vs. T2 (ref)			T1 vs. T3 (ref)	
	HR (95% CI)	P-value		HR (95% CI)	*p*-Value		HR (95% CI)	*p*-Value		HR (95% CI)	*p*-Value		HR (95% CI)	*p*-Value		HR (95% CI)	*p*-Value
Model 1	1.89 (0.98–3.62)	0.06		1.51 (0.81–2.82)	0.20		1.25 (0.67–2.35)	0.49		1.89 (0.95–3.77)	0.07		1.29 (0.66–2.49)	0.46		1.46 (0.75–2.85)	0.27
Model 2	1.85 (0.95–3.61)	0.07		1.51 (0.81–2.83)	0.20		1.22 (0.63–2.35)	0.55		1.76 (0.89–3.48)	0.10		1.43 (0.73–2.80)	0.30		1.24 (0.61–2.52)	0.56
Model 3	1.79 (0.89–3.60)	0.10		1.48 (0.77–2.85)	0.24		1.21 (0.62–2.37)	0.58		1.72 (0.85–3.51)	0.13		1.50 (0.73–3.06)	0.27		1.15 (0.56–2.38)	0.7

Model 1 was a minimally adjusted model with age, sex, Charlson comorbidity index, estimated glomerular filtration rate, geriatric nutritional risk index, and the categories of BMI change. Model 2 was adjusted for the same variables as model 1 in addition to the use of the mean blood pressure and angiotensin converting enzyme inhibitor/angiotensin II receptor blocker. Model 3 was adjusted for the same variables as model 2 in addition to the logarithmic brain natriuretic peptide and logarithmic C-reactive protein.

Abbreviations: BMI: body mass index; PD: peritoneal dialysis; ref: reference; HR: hazard ratio; CI: confidence interval.

A total of 57 (46.7%) patients experienced HF-related hospitalization. The median HF-related hospitalization-free survival time did not differ significantly between the T1, T2, and T3 groups (37.4 vs. 73.4 vs. 58.8 months, respectively; *p* = 0.19). In the adjusted Cox regression analysis, the risk of HF-related hospitalization tended to be higher in the T1 group than in the T2 group in models 1, 2, and 3 (HR: 1.89, 95% CI: 0.95–3.77, HR: 1.76, 95% CI: 0.89–3.48, and HR: 1.72, 95% CI: 0.85–3.51, respectively); however, there were no significant difference in this parameter neither between the T2 and T3 groups nor between the T1 and T3 groups (Table 4).

## Discussion

According to a previous report, the HR of death in patients on HD who lost weight (>5% BMI loss) was higher than that in those whose weights remained stable [14]. We hypothesized that PD might follow the trend of HD and investigated the association between the 6-month BMI change and death in patients on PD. Since withdrawal is a PD-specific problem, we also investigated the association between the BMI change and HD transfer in patients on PD. Consequently, this retrospective cohort study revealed patients on PD whose BMIs decreased (< −4.13%) from PD catheter insertion, the HRs of death and transfer to HD were higher than in those whose weights remained rather stable. This result was consistent with the findings of previous studies that a lower BMI leads to a worse prognosis in HD and PD [13]. On the other hand, the risk of HF due to volume overload and peritonitis, both major complications of PD [2,18], tended to be higher in patients on PD whose BMIs decreased than in those whose BMIs remained stable.

Although BNP and blood pressure at baseline were higher in the T1 group than in the T2 and T3 groups, there was no significant difference in either BNP or blood pressure at 6 months among the groups. This suggests that the higher BMI was indicative of fluid retention leading to increased blood pressure and that the improvement in fluid status due to PD induction may have been manifested as a decrease in the BMI. However, as a result, adjusting for BNP and MBP at baseline as confounders did not affect the results’ significance, suggesting that the results’ significance was not solely attributed to the change in fluid status, but also to the decrease in lean body mass and fat mass. To corroborate this assumption, when including IVCD instead of BNP at baseline as a fluid status marker, significantly higher HD transfer rate or all-cause mortality in patients in the T1 group compared to those in the T2 group persisted in a fully-adjusted model (HR: 2.41; 95% CI: 1.34–4.35). In any case, this study emphasizes that the 6-month BMI change is an unwavering prognostic factor, but at the same time, the limitation of BMI use should be recognized, as discussed in previous similar studies [13,14].

Thereafter, this study focused on PEW, which is closely associated with weight loss, especially the loss of lean body mass and fat mass, and may lead to worse outcomes in patients on PD. Indeed, there is room for PEW intervention, although a decrease in BMI due to fluid removal is inevitable, and appropriate intervention can prevent further weight loss. PEW is diagnosed based on at least three of four criteria: biochemical tests, BW, muscle mass, and dietary intake [28]. PEW and sarcopenia/frailty are bidirectionally related, as a decrease in the muscle mass is associated with the diagnosis of sarcopenia, and weight loss is associated with the diagnosis of frailty. Patients with CKD are prone to inadequate nutrition, inflammation, acidosis, and endocrine disturbances, leading to increased proteolysis and lipolysis. In other words, they tend to be prone to PEW conditions [29]. PEW is also related to physical frailty, cardiovascular disease, and poor quality of life; it is a risk factor for mortality [30]. Although not significant, probably due to the limited sample size, HF-related hospitalization and peritonitis tended to be more common in the T1 group, suggesting that the patients in the T1 group developed PEW, which increased their susceptibility to HF and peritonitis, leading to an increase in the rate of HD transfer or all-cause mortality. Actually, the proportion of HF as a cause of HD transfer was significantly higher in the T1 group than in the T2 group. Therefore, it is necessary to take measures to avoid PEW and maintain the BMI at > −4.13% from catheter insertion in patients on PD, as these two sets of measures may be independently associated with a favorable prognosis.

To prevent PEW and achieve a stable or increased BMI, adequate energy intake (25–35 kcal/kg/day) and protein requirement (1.0–1.2 g/kg/day) are recommended in patients on HD and PD [31,32]. Minimal energy intake accelerates protein catabolism and low protein intake (<0.8 g/kg/day) has been reported to be associated with an increased mortality rate [33]. The CKD guidelines recommend Medical Nutrition Therapy recommendations by a registered dietitian/nutritionist, physician, or nursing practitioner to assess adequate caloric intake and protein. They also state 24-h food recalls and food frequency questionnaires [31]. Considering that CKD patients are more prone to PEW, the appropriate treatment for metabolic disorders such as metabolic acidosis, chronic inflammation, and hormone deficiencies, as well as the prescription of an optimized dialysis regimen, are also necessary [29].

Additionally, the BW/BMI was significantly higher in the T1 group than in the other groups. Actually, in patients on PD with high BMIs, it is often difficult to consistently achieve an adequate Kt/V over time despite a greater increase in the rate of PD prescription because of the higher total body fluid volume, which may predispose patients with a high BMI to PD failure and metabolic abnormalities [25]. However, the significant association between the longitudinal decrease in the BMI and the worse PD-related outcomes persisted after adjusting for GNRI (calculated from the BMI) as a confounder. This significant association was persistent even after direct adjustment for baseline BMI as a covariate, indicating that the poor prognosis of patients in the T1 group cannot be fully explained by a higher baseline BMI per se; however, careful follow-up is required for patients with a high BMI because they may be more likely to experience a subsequent decline in BMI. Moreover, the baseline BNP was repeatedly higher in the T1 group than in the T2 group, suggesting the fluid overload in the T1 group; however, BNP was not significantly different among the three tertile groups at 6 months. Therefore, it seems unlikely that the overhydrated status at baseline in the T1 group remained and directly led to increased HD transfer or all-cause mortality, particularly from fluid overload.

Our study had several limitations. The first and major limitation was that we focused only on the change in the BMI. Certainly, the BMI is an important indicator of the body’s size; however, it includes the lean body mass, fat mass, and fluid mass. Since the BMI does not distinguish between the lean body mass, fat mass, and fluid mass, it is undeniable that a change in the fluid volume may have contributed to our conclusion. At the same time, we included BNP as a marker of volume overload to be adjusted for in multivariate models to minimize the effect of changes in the fluid status. In addition, bioelectrical bioimpedance analysis (BIA) was not performed to assess fluid status routinely during the whole study period. However, the BIA data is not perfect to assess fluid status, as is can be affected by the dialysate fluid in the peritoneal cavity, hypoalbuminemia, and muscle wasting, and a clear superiority to BNP or IVCD on predicting outcomes in patients with PD has not been elucidated [34]. In recent years, the trajectory of the lean body mass and fat mass has attracted attention in HD [35]. Therefore, future studies to investigate the association between the lean body mass, fat mass, and fluids trajectory and death or HD conversion in patients on PD were warranted. Second, the participants’ race and socioeconomic statuses were neither investigated nor analyzed. However, because we recruited only Japanese participants in this study, the patients’ backgrounds were relatively similar. Third, this study was both an observational cohort study and a single-center study with a limited sample size. Although we performed multivariate analyses using several models to minimize the effects of covariates, we could not eliminate the influence of other potential confounding factors. Finally, because we did not perform and record the PD adequacy test routinely during the whole study period, the data regarding ultrafiltration, Kt/V, and creatinine clearance could not be obtained, which might affect patient outcome. Conversely, various anthropometric and biochemical data at 6 months have been collected and these parameters have been clarified, including D/P4, were equivocal among the three tertile groups, suggesting that PD adequacy was similar among the groups.

## Conclusion

In conclusion, a decrease in the BMI over time is independently associated with PD discontinuation or all-cause mortality among patients initiating PD. Our study emphasizes the importance of the BMI decrease as a novel prognostic marker. To investigate interventions, including BMI optimization, further multi-center large-scale studies are needed as a novel therapeutic target to improve the time on PD therapy and patients’ survival.

## Supplementary Material

Supplemental Material
