# Supplementary material for: The body mass index change is associated with death or hemodialysis transfer in Japanese patients initiating peritoneal dialysis
Source: Ren Fail. 2023 Jan 13;45(1):2163904. doi: 10.1080/0886022X.2022.2163904 (PMC9848317; doi:10.1080/0886022X.2022.2163904)
Supplement: Supplemental Material [file IRNF_A_2163904_SM6412.pdf]

Table S1: The study population’s anthropometric and biochemical variables at 6 months after baseline and the groups divided according to body mass index change (T1: BMI change: <−4.13%, T2: BMI change: −4.13–0.67%, and T3: BMI change: >0.67%)

Variables	Total (n = 122)	T1 (n = 41)	T2 (n = 40)	T3 (n = 41)	P-value
Systolic blood pressure (mmHg)	136.8 ± 17.5	138.1 ± 18.3	134.0 ± 13.8	138.3 ± 19.7	0.5
Diastolic blood pressure (mmHg)	78.1 ± 12.5	77.9 ± 11.8	78.9 ± 10.4	77.5 ± 15.1	0.77
Mean blood pressure (mmHg)	96.1 ± 17.6	97.9 ± 12.7	94.8 ± 18.2	95.3 ± 21.2	0.7
Body weight (kg)	63.9 ± 14.5	64.9 ± 14.8	61.1 ± 12.9	65.6 ± 15.6	0.38
BMI (kg/m^2)	23.1 (21.0–25.9)	23.2 (21.1–26.6)	22.7 (20.4–24.8)	23.4 (21.6–27.6)	0.16
IVCD (cm) (n = 119)	1.6 ± 0.4	1.5 ± 0.4	1.5 ± 0.3	1.7 ± 0.4	0.03
Albumin (mg/dL)	3.3 (3.0–3.7)	3.2 (2.9–3.6)	3.4 (3.2–3.7)	3.3 (3.0–3.7)	0.18
Urea (mg/dL)	59.7 (51.7–69.9)	61.4 (51.6–69.5)	60.1 (53.3–68.8)	58.1 (50.5–72.8)	1
Creatinine (mg/dL)	9.1 (7.9–11.6)	9.5 (8.2–11.4)	8.7 (7.9–10.8)	9.2 (7.0–11.8)	0.57
eGFR (mL/min/1.73m^2)	5.0 ± 1.5	4.9 ± 1.7	5.0 ± 1.2	5.0 ± 1.6	0.57
Hemoglobin (g/dl)	10.2 ± 1.1	10.2 ± 1.1	10.2 ± 0.9	10.2 ± 1.2	0.67
Calcium(mg/dL)	8.4 ± 0.7	8.4 ± 0.7	8.4 ± 0.6	8.3 ± 0.7	0.63
Phosphorus (mg/dL)	5.4 (4.8–6.0)	5.7 (4.9–6.1)	5.0 (4.8–6.0)	5.3 (4.8–5.8)	0.33
PTH (pmol/l)	229 (131–351)	224 (111–326)	229 (118–325)	231 (153–416)	0.4
CRP (mg/L)	0.07 (0.02–0.25)	0.08 (0.02–0.34)	0.07 (0.02–0.19)	0.07 (0.02–0.21)	0.83

BNP (pg/mL)	72.4 (32.8–157.1)	69.8 (37.3–147.3)	55.3 (23.0–103.1)	100.7 (60.0–244.2)	>0.05
GNRI	94.1 ± 10.3	92.8 ± 10.8	93.7 ± 8.7	95.8 ± 11.2	0.41
D/P4 (n = 101)	0.69 ± 0.14	0.68 ± 0.15	0.67 ± 0.14	0.70 ± 0.13	0.67

P-values were estimated by the one-way analysis of variance and the Kruskal–Wallis test for normally and non-normally distributed continuous variables, respectively.

Abbreviations: SD, standard deviation; BMI, body mass index; IVCD, inferior vena cava diameter; eGFR, estimated glomerular filtration rate; PTH, parathyroid hormone; CRP, C-reactive protein; BNP, brain natriuretic peptide; GNRI, geriatric nutritional risk index; D/P4, dialysate-to-plasma ratio of creatinine at 4 h.

Table S2. Association between BMI change and death or HD transfer in patients on PD using standard Cox regression model in sensitivity analyses

HD transfer or all-cause mortality	T1 vs. T2 (ref)		T3 vs. T2 (ref)		T1 vs. T3 (ref)	
	HR (95% CI)	P-value	HR (95% CI)	P-value	HR (95% CI)	P-value
Model 1’	1.99 (1.20–3.30)	0.008	1.10 (0.65–1.84)	0.73	1.83 (0.98–3.41)	0.06
Model 2’	2.02 (1.20–3.40)	0.008	1.09 (0.65–1.83)	0.74	1.86 (0.98–3.52)	0.06
Model 3’	1.70 (1.02–2.83)	0.04	0.78 (0.43–1.40)	0.41	2.20 (1.14–4.26)	0.02

Model 1’ was a minimally adjusted model with age, sex, Charlson comorbidity index, estimated glomerular filtration rate, logarithmic BMI, serum albumin level, and the categories of BMI change. Model 2’ was adjusted for the same variables as model 1’ in addition to the use of the mean blood pressure and angiotensin converting enzyme inhibitor/angiotensin II receptor blocker. Model 3’ was adjusted for the same variables as model 2’ in addition to the logarithmic brain natriuretic peptide and logarithmic C-reactive protein.

Abbreviations: BMI, body mass index; HD, hemodialysis; PD, peritoneal dialysis; ref, reference; HR, hazard ratio; CI, confidence interval
